# Mindboggle: Automated brain labeling with multiple atlases

**DOI:** 10.1186/1471-2342-5-7

**Published:** 2005-10-05

**Authors:** Arno Klein, Brett Mensh, Satrajit Ghosh, Jason Tourville, Joy Hirsch

**Affiliations:** 1 fMRI Research Center, Columbia University, New York, USA; 2 Parsons Institute for Information Mapping, The New School, New York, USA; 3 New York State Psychiatric Institute, Columbia University, New York, USA; 4 Speech Communication Group, Research Laboratory of Electronics, Massachusetts Institute of Technology, Cambridge, USA; 5 Department of Cognitive and Neural Systems, Boston University, Boston, USA

## Abstract

**Background:**

To make inferences about brain structures or activity across multiple individuals, one first needs to determine the structural correspondences across their image data. We have recently developed Mindboggle as a fully automated, feature-matching approach to assign anatomical labels to cortical structures and activity in human brain MRI data. Label assignment is based on structural correspondences between labeled atlases and unlabeled image data, where an atlas consists of a set of labels manually assigned to a single brain image. In the present work, we study the influence of using variable numbers of individual atlases to nonlinearly label human brain image data.

**Methods:**

Each brain image voxel of each of 20 human subjects is assigned a label by each of the remaining 19 atlases using Mindboggle. The most common label is selected and is given a confidence rating based on the number of atlases that assigned that label. The automatically assigned labels for each subject brain are compared with the manual labels for that subject (its atlas). Unlike recent approaches that transform subject data to a labeled, probabilistic atlas space (constructed from a database of atlases), Mindboggle labels a subject by each atlas in a database independently.

**Results:**

When Mindboggle labels a human subject's brain image with at least four atlases, the resulting label agreement with coregistered manual labels is significantly higher than when only a single atlas is used. Different numbers of atlases provide significantly higher label agreements for individual brain regions.

**Conclusion:**

Increasing the number of reference brains used to automatically label a human subject brain improves labeling accuracy with respect to manually assigned labels. Mindboggle software can provide confidence measures for labels based on probabilistic assignment of labels and could be applied to large databases of brain images.

## Background

When comparing structures or functions across brains, it is common to label the gross anatomy of brain image data and to compare the structures or functions that lie within anatomically labeled regions. Since brains differ in their anatomy [1-10], it would seem reasonable to refer to the anatomy of many brains when labeling an individual subject's brain image. Atlases are manually labeled brains used as references. Using every atlas from a group of atlases independent of each other was found to give labeling results superior to those obtained by selecting the closest matching single atlas from the group, the average atlas, or an individual atlas, for the case of confocal microscopy images of bee brains [11]. However, labeling a subject's brain image with many different brains presents unreasonable demands on human labelers, who may not be consistent in their label assignments [12-15]. Fully automated labeling would facilitate large-scale labeling efforts while adding efficiency and consistency.

Image registration software (reviewed in [16-18]) may be used to coregister subject and atlas brain images, thereby labeling the subject images with superimposed atlas labels. There exist many different nonlinear image registration and feature-matching approaches to this problem [19-62]. Mindboggle software (see below) offers certain advantages over most of these approaches: it does not make the same assumptions about preserving topography from brain to brain, is relatively fast, and it performed well in comparison tests with standard image registration software packages (AIR, SPM2, ANIMAL, and linear registration with FLIRT) and in artificial lesion tests [63].

Having an automated registration or feature-matching program and a database of atlases introduces the problem of how to reconcile the multiple atlas label sets when labeling a single subject's brain. Labels could be assigned based on the selection or construction of similar or representative anatomy from these atlases. It is becoming more common to label subject brain image data with a single, composite atlas representing some average of multiple brain atlases (an average brain atlas) or retaining information about the differences between the atlases or between the atlases and the subject brain image (a probabilistic brain atlas).

Average brain atlases attempt to assign to each voxel (volume element) a representative value associated with image intensity or anatomical label. An intensity-based average brain atlas is the voxelwise mean intensity across individual brain images after linear [64-66] or nonlinear [67] coregistration. Additionally or alternatively, an average brain atlas may represent average sulcus shapes and positions computed in the original brain image space [8,68,69] or in an alternative space such as on a sphere [70]. An example of a label-based average brain atlas was constructed by Hammers, et. al. [71], where the majority label was computed for each voxel across 20 manually labeled brains after nonlinear registration to the MNI152 [64] template using SPM99 [27]. The use of an average atlas presupposes that there is such a thing as a representative brain and does not usually account for variability across brains.

Probabilistic brain atlases, on the other hand, do provide additional statistical information across the population used to construct the atlas [62,72-82]. This information may be related to the variance of landmark positions [73], probability of anatomical labels [44,79,83,84], probability of tissue classes [80], or multiple anatomical dimensions, for example characteristics of surface geometry and Bayesian priors associated with neighborhood relations between labels [62], and the multi-dimensional atlases under development by Mazziotta and Zilles and their colleagues [72,77,78,81]. An abstract representation of a database of manually labeled brains can also serve as a probabilistic atlas; for example, expert neural networks trained on a learning database of such brains [48] or graphs relating parametric surfaces [36]. However, there are only two examples known by the authors in which a complete cortical atlas is constructed from multiple label sets where each label set was assigned manually [62,71], rather than by automating the labeling of many brains without independent validation of the labeling technique. As with average brain atlases, probabilistic atlases have primarily been used as templates to which a subject brain is transformed and compared. This comparison presupposes that the single transform will account for differences between the subject brain image and each of the multiple brain images that were used to construct the atlas.

In this paper, we have chosen to extend the use of an individual atlas to multiple atlases in a recently introduced, fully automated, feature-based nonlinear labeling method called Mindboggle (freely downloadable, open source Matlab code) [63,85]. Rather than use a single (average or probabilistic) atlas, Mindboggle employs each atlas in a database independently to label the cortical voxels of a subject brain image, and for each voxel chooses the majority label assigned by the different atlases. We explore the effects of using two different labeling schemes and variable numbers of atlases on labeling accuracy and on the numbers of labels assigned per voxel.

## Methods

### Image acquisition

We used two sets of T1-weighted MRI data from a total of 20 young, healthy adult subjects. The first group of 10 subjects was scanned at the MGH/MIT/HMS Athinoula A. Martinos Center for Biomedical Imaging using a 3T Siemens scanner and standard head coil (TE: 2.9 ms, TR: 6.6 ms, flip angle: 8°). The in-plane resolution was approximately 1 × 1 mm, the slice thickness was 1.33 mm, and the dimensions and field of view were 256 × 256 voxels. These subjects consist of four men and six women between the ages of 22 and 29 years old (*μ *= 25.3). All are right-handed. The data were bias-corrected, affine-registered to the MNI152 template [64], and segmented using SPM2 software [27].

The second group of 10 subjects was scanned at Columbia University on a 1.5T GE scanner (TE: 5 ms, TR: 34 ms, flip angle: 45°). Slice thickness was 1.5-mm axial, in-plane resolution was 0.86 mm. Images were resliced coronally to a slice thickness of 3 mm, rotated into cardinal orientation, then segmented and parcellated using Cardviews software from MGH. These subjects consist of five men and five women between the ages of 26 and 41 years old (*μ *= 32.7).

### Image processing before applying Mindboggle algorithm

Mindboggle calls on third-party software to perform three preliminary steps on a subject brain image: (1) cropping non-brain matter, (2) linear coregistration with the MNI152 template [64], and (3) segmentation into gray matter, white matter, and cerebrospinal fluid. For this study, these steps were performed by (1) BET [90], (2) FLIRT [91] set to correlation ratios, 12-parameter affine transforms and trilinear interpolation, and (3) SPM2 [27] for the first group of 10 brains and FAST [92] for the second group of 10 brains.

### Mindboggle algorithm

Mindboggle is a freely downloadable, open source software package written in Matlab (version 6, release 13, with the Image Processing Toolbox, The Mathworks Inc., USA) and has been tested on different models of desktop and laptop computers running different distributions of Linux, as well as MacOSX and Windows. The general system requirements are the basic requirements of the Matlab environment. The system used to conduct the following tests consists of a 2.2 GHz Pentium IV processor running Redhat Linux 9.0 on a PC with 1 GB memory. Mindboggle was selected as the nonlinear method because it was created by one of the authors (AK) and performed favorably in comparisons with the popular nonlinear methods AIR, ANIMAL, and SPM2 [63].

**Figure 1:**
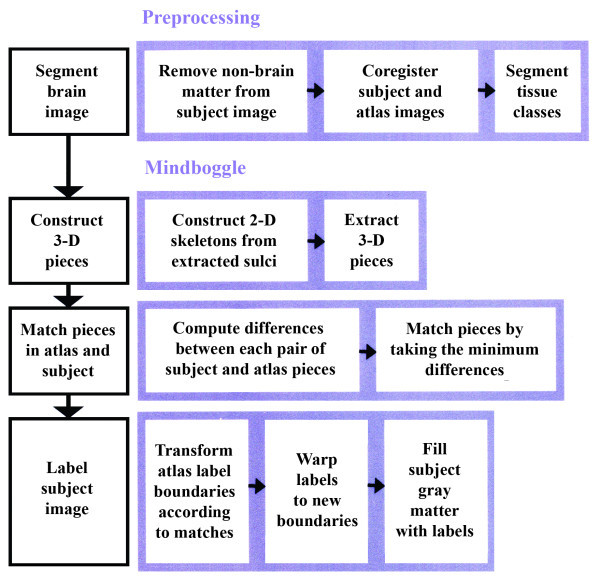
Mindboggle flowchart.

Mindboggle's general strategy is to fill a subject's cortical gray matter mask with atlas labels, based on correspondences found between structures in a subject image and in one or more atlases (see Figure 1). Details of the original algorithm may be read in [63], and consist of the following five steps performed on a subject's brain image data:

If we divide the voxels into groups, by the number of different labels per voxel, as in Figure 10, we may see that there is an inverse relationship between the number of different labels and the label agreement with manual labels. Therefore, the number of labels per voxel provides a rough confidence measure for the majority label assigned to each voxel.

(1) extract cerebral cortical sulci,

(2) prepare hundreds of pieces from image-processed versions of these sulci,

(3) match each piece from an atlas with a combination of pieces from the subject,

(4) translate local atlas label boundaries according to the difference in position between each match, and

(5) warp the atlas label volume to the transformed boundaries and propagate these labels to fill a subject mask. Mindboggle optionally resets planar boundaries for frontal and temporal poles as well as the occipital lobes, if the atlas itself is labeled using these planar boundaries.

Mindboggle extracts cerebral cortical sulci in the following manner (see Note 1 in Appendix). First, Mindboggle crops exposed brain surface by eroding the segmented cortex three voxels deep. Mindboggle also crops subcortex and cerebellum with a mask constructed from a union of two of the Montreal Neurological Institute's atlases: the single-subject atlas [93] and the MNI152 template [64]. All registration and labeling by Mindboggle is performed in MNI152 space (resolution of 1 × 1 × 1 mm and dimensions of 181 × 217 × 181 voxels).

**Figure 2:**
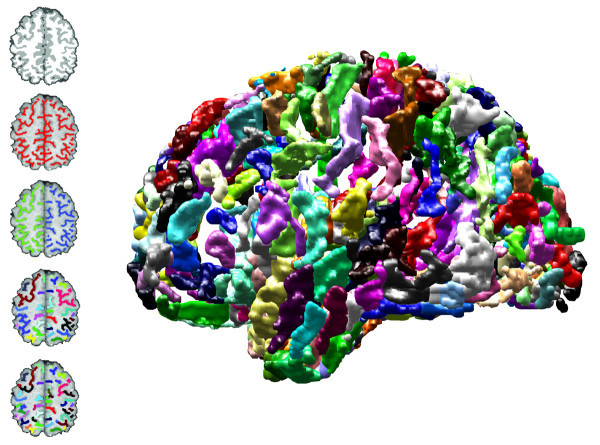
**Piece construction in Mindboggle**. Shown on the left, from top to bottom, are the five steps Mindboggle takes to construct pieces from a subject brain image. Darker pixels (non-white matter) of a (1) segmented horizontal slice are (2) thinned to a skeleton which is (3) split into left and right hemispheres. (4) Contiguous pixels of the skeleton slice are grouped into 2-D pieces, and (5) these 2-D pieces are used to construct 3-D pieces, shown in cross-section (bottom) and in 3-D (right figure, showing the left side of the brain with the frontal pole facing left).

Sulcus pieces are constructed as follows (see Figure 2). The segmented gray matter with cerebrospinal fluid is thinned to a pixel-wide skeleton for each slice (Matlab's bwmorph.m function). All of the skeletonized slices are stacked to create a 3-D skeleton. This skeleton is split by an interhemispheric plane formed by warping a vertical plane to the medial slab of the skeleton using a modified Self-Organizing Map algorithm (see Note 2 in Appendix). The skeleton is then broken up into pieces as follows. Starting from the top slice of the skeleton, each set of connected pixels is considered a separate piece. Each pixel in the slice below is assigned membership to the nearest piece in the above slice. The latter operation is repeated from top to bottom, as well as from bottom to top, resulting in two independent sets of candidate pieces, with each pixel having two assignments, one for each set. A single set of pieces is obtained by identifying the unique set of pairs of assignments. The 3-D pieces are then fragmented using a k-means algorithm and regrouped together if they share extensive borders. This last regrouping step is conducted so that compact structures with a low surface-to-volume ratio such as a ball do not get broken up in arbitrary ways by the k-means algorithm. "Extensive borders" is defined as a ratio of border to surface voxels equal to at least one-tenth, where a border voxel has at least one other piece in its immediate neighborhood of six voxels, and a surface voxel has fewer than six occupied voxels in its neighborhood.

Finding similar pieces in an atlas helps to determine how to transform atlas label boundaries, and therefore how to distribute atlas labels in the subject brain. Matching each piece from an atlas with a combination of (up to three) similar pieces from the subject is performed by minimizing a cost function. The cost function consists of a sum of normalized quantities derived from differences in: mean position, number of voxels, number of subvolumes, and non-overlap. Differences in mean position and number of voxels are measures of the differences in location and size between the atlas and subject pieces. The number of subvolumes for a given piece is the number of 5 × 5 × 5-voxel boxes dividing the image volume that contain the piece. This measure is useful for distinguishing between pieces that have different spatial distributions, such as between a tight ball and an extensive sheet. Non-overlap of two pieces, P1 and P2, is equal to the fraction of subvolumes of P1 that do not overlap P2 added to the fraction of subvolumes of P2 that do not overlap P1. This measure is useful for distinguishing between differently shaped pieces that may otherwise be similar according to the other three measures.

Atlas label boundaries are locally translated according to the difference in position between nearby atlas and matching subject pieces. The translation is the difference of the mean of the local boundary from the mean of the subject piece(s), plus the difference between the mean of the atlas piece from the mean of the local boundary (after scaling by the ratio of the atlas and subject piece bounding boxes).

The atlas label volume is then warped to the transformed atlas label boundaries as follows (see Note 3 in Appendix). The atlas label that was closest to each original boundary point moves to the transformed boundary point, carrying along its neighboring labels as a function of their distance from the point (according to a Gaussian distribution function). After warping, each unlabeled voxel within the segmented gray matter mask is assigned the majority label in its 5 × 5 × 5-voxel neighborhood; this last step is repeated several times.

### Evaluation

We evaluated labels assigned by Mindboggle to a brain image (in MNI152 space) by comparing them with the manual labels for that brain (linearly registered to MNI152 space). The manual labels used for evaluation were also used to construct Mindboggle atlases. They were assigned by a single human labeler to each of the 20 subject brains (before linear registration to the MNI152 space), according to one of two different parcellation schemes. The first group of 10 subjects was labeled by Jason Tourville according to a scheme that is a modified version of Cardviews (see below) and implemented in a software tool developed by Satrajit Ghosh at the Department of Cognitive and Neural Systems, Boston University [94]. The second group of 10 subjects was labeled by Olga Kambalov according to the Cardviews parcellation scheme, created at the Center for Morphometric Analysis, Massachusetts General Hospital, and implemented in Cardviews software [12]. The labeler for each group of subjects is an expert in Cardviews.

For both parcellation schemes, 74 cortical labels were selected from the original 96 labels and merged to give 36 labels (18 per hemisphere): superior, middle, and inferior frontal and temporal gyrii, frontal and temporal poles, pre- and postcentral gyrii, superior and inferior parietal lobules, occipital lobe, fusiform, lingual/parahippocampal, and orbital (frontal) gyrii, insula, and cingulate gyrus. The anatomical divisions are coarser than those of Cardviews primarily because regions divided by planes in the Cardviews approach are combined.

**Figure 3:**
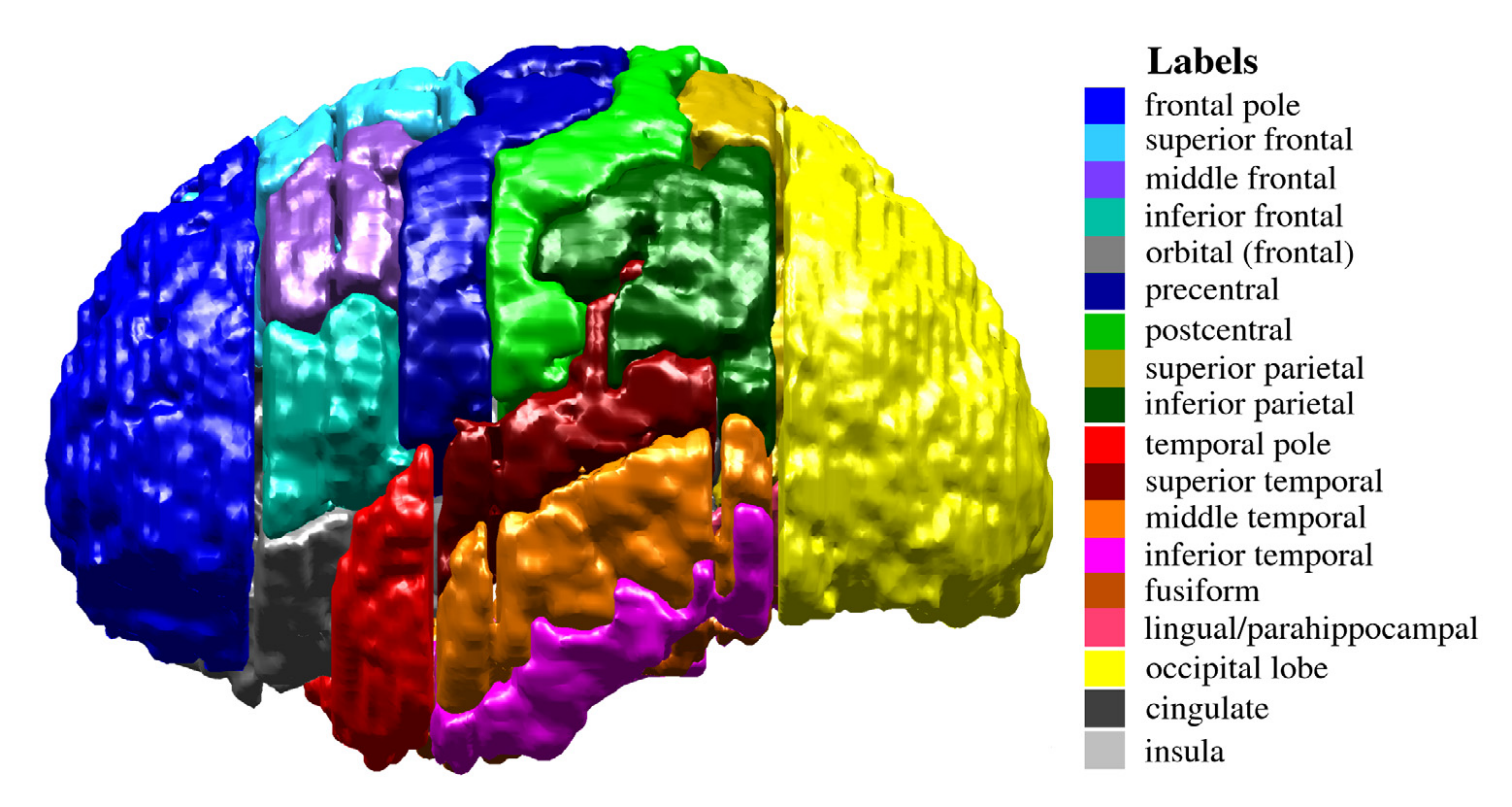
**Manual labels**. Manual labels for a single subject (left side, frontal pole facing left), drawn from the first subject pool (modified Cardviews labels). After these labels are registered to the common (MNI152) space, they are processed to construct one of the atlases for labeling with Mindboggle. This figure is an isosurface representation constructed with a Gaussian filter of radius three voxels. Missing data in vertical strips are due to incompletely labeled coronal sections.

Figure 3 presents an isosurface representation of a single manually labeled subject brain. To determine whether increasing the number of atlases would improve the accuracy of Mindboggle labeling, we compared the manual label for each voxel of a subject image with the majority of all Mindboggle labels for that voxel, for an increasing number of atlases used to assign labels. When determining the majority label, ties were broken by random selection. Each subject is automatically labeled by a random selection of atlases for each number of atlases. For comparisons up to nine atlases, the atlases were randomly selected from within the same subject pool; for comparisons up to 19 atlases, atlases were randomly selected from either subject pool.

The primary evaluation measure we employ is percent label agreement between atlas labels and manual labels assigned to a subject's segmented gray matter mask, with each gray matter voxel having one manual and one automated (Mindboggle) label. The agreement between atlas label set *A*_*i *_and manual label set M_*i *_is defined as the volume of intersection divided by the volume of the manually labeled region, computed in voxels and summed over a set of multiple labeled regions each with index i, where |.| indicates number of voxels:



Our type I error, a measure of how many incorrect labels are found in a given manually labeled region, is simply equal to one minus the label agreement for that region. We define a type II error for a given manually labeled region as the number of automatically labeled voxels outside the region that have been assigned that region's label, divided by the total number of automatically labeled voxels with that label. This is equal to one minus the fraction of voxels automatically assigned a given label that lies within the corresponding region:



These error measures assume that the manual labels are correct, and they can range from zero to one; a value of zero is achieved when automated and manual labels perfectly overlap for each label.

Another evaluation measure we employ is percent label accord [12], the intersection between two similarly labeled regions divided by the mean volume of the two regions:



The above voxel-based measures ignore misregistration within a labeled region. Any conclusions based on them must therefore be restricted to the labeled volumes and may not be applicable to finer resolutions.

## Results and discussion

**Figure 4:**
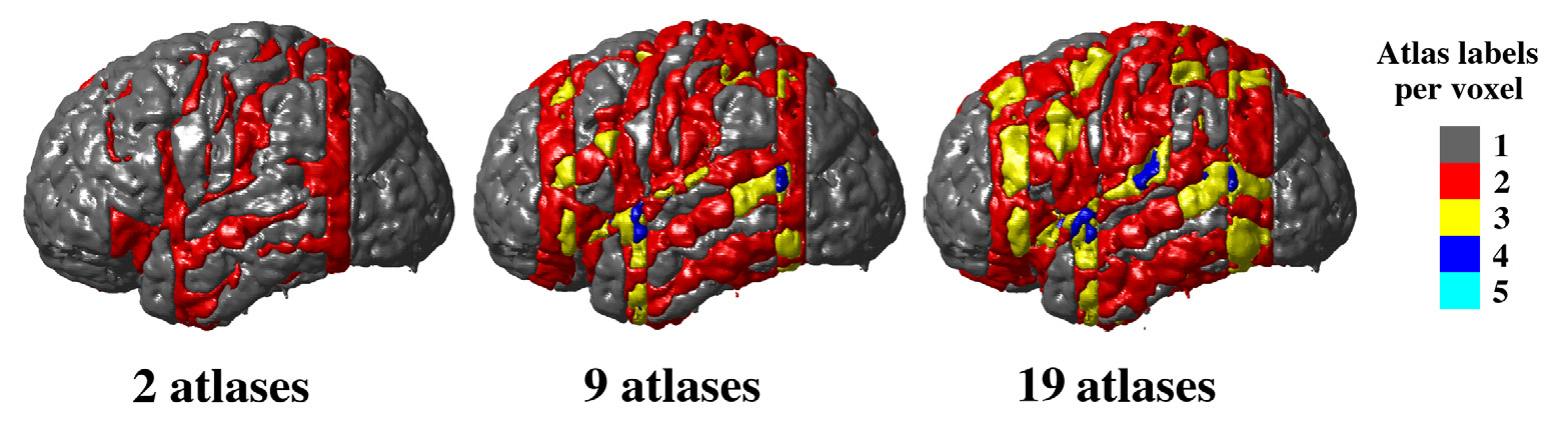
**Spatial distribution of the number of labels per voxel**. The isosurface representations of this subject are colored to indicate the number of different labels assigned to each voxel by the different atlases (for example, gray indicates one label, when all atlases agree). From left to right, each brain has been labeled by Mindboggle using an increasing number of atlases (2, all 9 from the same subject group, and all 19 from both subject groups). As one would expect, increasing the number of atlases inceases the average number of different labels assigned to each voxel. Missing data in vertical strips are a result of incompletely labeled coronal sections, as in Figure 1. The data for all subjects and for every number of atlases are graphed in Figure 3.

**Figure 5:**
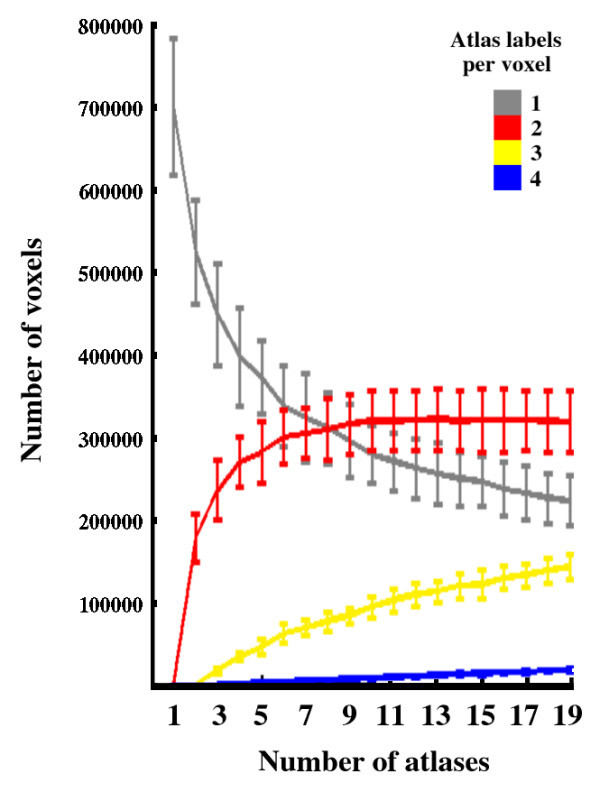
**Quantity of voxels with a given number of labels per voxel**. This is a graph of all the subject data labeled with an increasing number of atlases, from which the single subject in Figure 2 was drawn. The total quantity of labeled subject voxels, representing the total volume of labeled gray matter, was around 700,000 voxels on average. Remaining data for voxels with five or more atlas labels were not included for clarity.

We found there to be greater disagreement between atlases as the number of atlases increases, as one would expect. This is clearly demonstrated in Figures 4 and 5. Figure 4 displays the anatomical distribution of the number of different labels assigned by the atlases to each voxel. Figure 5 plots the total number of voxels with a given number of different labels per voxel. Both figures present their data as a function of the number of atlases. If we compare Figure 4 with Figure 3 (it is the same subject), the disagreements are clustered about anatomical boundaries, with the highest numbers of labels per voxel at the boundaries between multiple anatomical regions, as one would expect.

**Figure 6:**
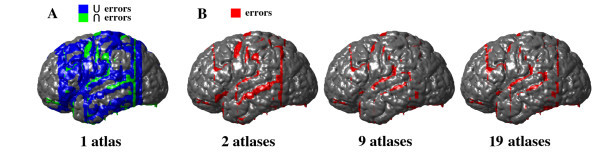
**Labeling errors**. Fig. 4A demonstrates the variability in the spatial distribution of labeling errors for a single atlas labeling a subject, across all atlases. Blue indicates voxels where at least one atlas disagrees with the subject's manual labels (union). Green indicates voxels where every atlas disagrees with the subject's manual labels (intersection). Fig. 4B demonstrates the effect of the use of multiple atlases on labeling errors. Red voxels are those whose manually assigned label disagrees with the majority of the labels assigned by Mindboggle using multiple atlases. If we look from left to right, we see that increasing the number of atlases reduces labeling errors. Atlas selection and isosurface representation match the conditions of Figure 2.

**Figure 7:**
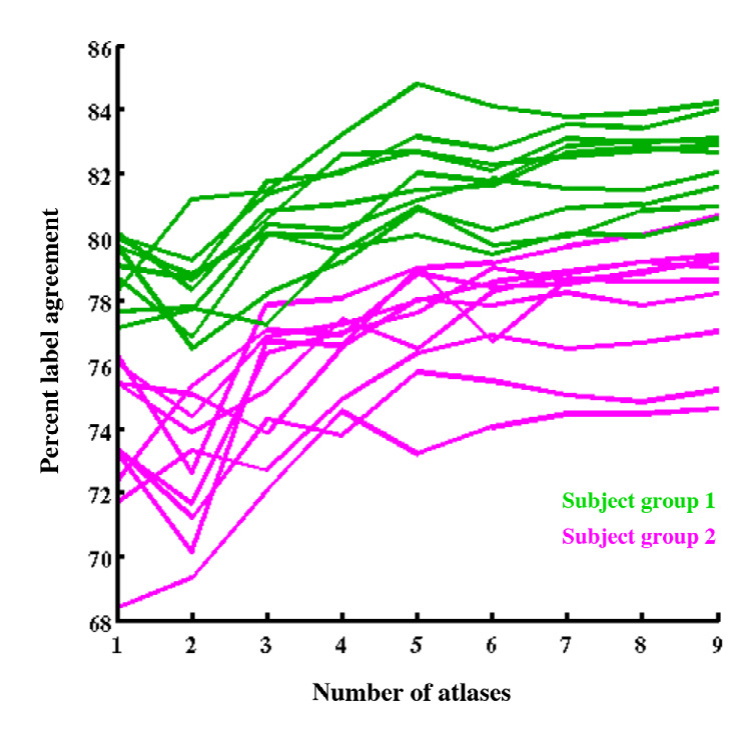
**Percent label agreement by subject pool**. The two subject groups are manually labeled with slightly different parcellation schemes. Each member from the first group of 10 subjects (green) was labeled with one atlas from the same group, then two, three, up to nine atlases, with each atlas selected at random from the remaining unselected atlases. The same procedure was repeated for each member from the second group of 10 subjects (magenta). As may be seen here, the percent label agreements obtained by Mindboggle are clearly separable between the two groups. Therefore, Mindboggle is sensitive to variance in the subject population, and to the parcellation scheme used to manually label the atlases.

Figure 6A demonstrates the variability in labeling errors when different single atlases are used to label one subject. Figure 6B demonstrates the effect of the use of multiple atlases on labeling errors for the same subject. Figure 6B indicates that increasing the number of atlases reduces labeling errors in Mindboggle. Figure 7 demonstrates that the two subject populations, manually labeled with slightly different parcellation schemes, give clearly separable labeling results, and that the label agreement between manual labels and (voxelwise majority) Mindboggle labels remains distinct between the two subject groups even as the number of atlases increases. Each member from the first group of 10 subjects was labeled with one atlas from the same group, then two, three, up to nine atlases, with each atlas selected at random from the remaining unselected atlases. The same procedure was repeated for each member from the second group of 10 subjects. For each member of the combined subject population, one to 19 atlases are selected at random from either subject group.

Therefore, Mindboggle is sensitive to variance in the subject population and to the parcellation scheme used to manually label the atlases, in particular to the vertical planes that are used to define boundaries to large regions (occipital lobe, frontal and temporal poles). These planes are not positioned by the sulcus piece matching stage but by an automated identification and matching of specific anatomical landmarks. The definitions of these landmarks may be different between parcellation schemes and may not be as consistently or as accurately determined manually or automatically in one scheme versus another. Some of the differences between the results obtained by the two subject groups (see Methods: Image Acquisition) may be attributed to the broader sampling in the second group of subjects (three races versus one, unknown vs. right-handed, and much wider age range). We can expect even greater deviations from brains that are very young, very old, or inflicted with a pathological condition, something we are presently investigating.

**Figure 8:**
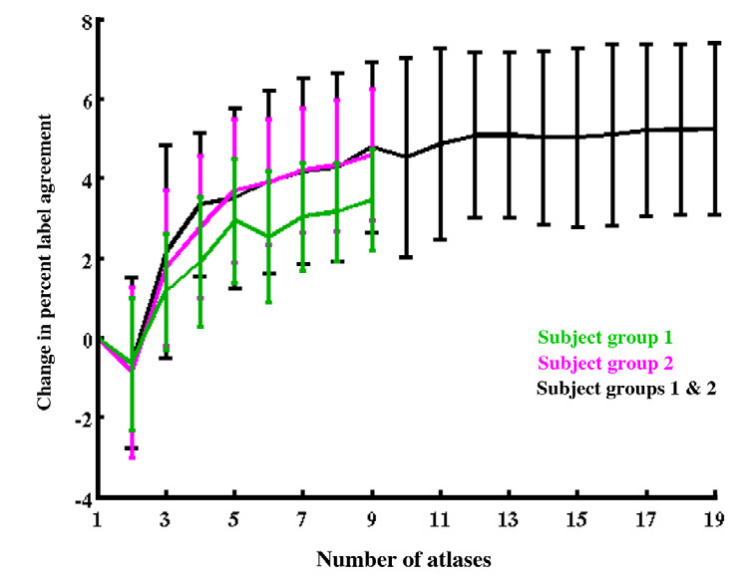
**Change in label agreement as a function of the number of atlases**. Increasing the number of atlases results in an asymptotic increase in the mean label agreement between labels assigned manually and by Mindboggle. The error bars extend one standard deviation about the mean. Data from the first group of subjects alone are in green and from the second group alone are in magenta. Data from both groups, where one to 19 atlases are selected at random from either subject group, are in black. Table 1 contains the data used in this figure.

**Figure 9:**
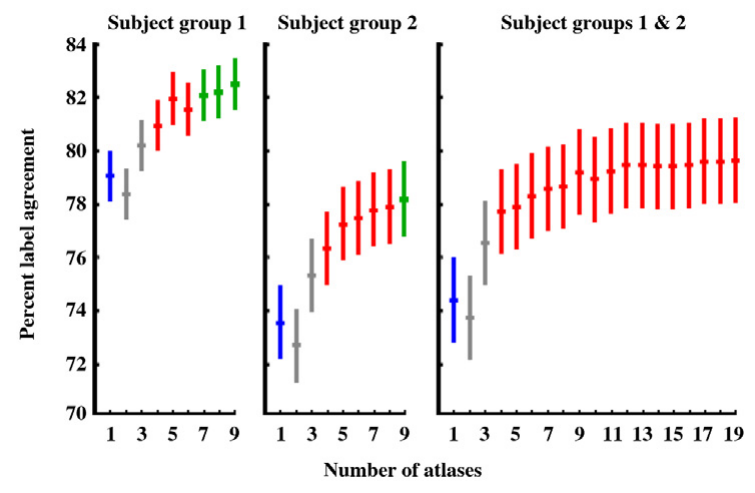
**Comparison between label agreements obtained with different numbers of atlases**. A one-way ANOVA was performed to test if the means are the same for the label agreements obtained by the different numbers of atlases. A multiple comparison test was then performed using Tukey's honestly significantly difference criterion to determine which pairs of means are significantly different. The graph displays the mean for each number of atlases with a 95% confidence interval around the mean, based on the Studentized range distribution. If intervals are disjoint, their means are considered significantly different. The label agreement obtained with a single atlas is in blue and any significantly different result is in red or green. Green results are significantly higher than gray results (using three atlases). Using at least four atlases resulted in significantly higher label agreements and lower type II errors than when using one atlas (*p *≪ 10^-6^), suggesting that Mindboggle should be used with at least four atlases to benefit from the multiple atlas approach.

**Figure 10:**
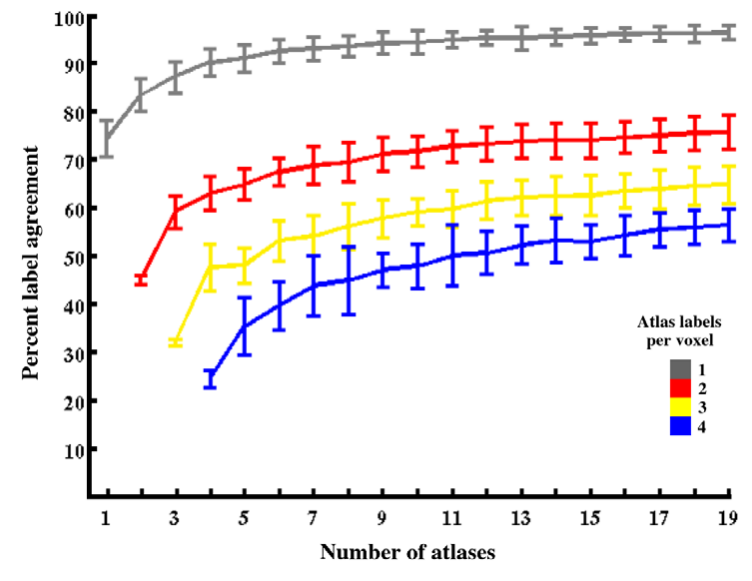
**Label agreement for different numbers of atlases and labels per voxel**. The data of Figure 6 are broken up here into subsets of voxels according to the number of different atlas labels assigned to each voxel. Voxel populations with fewer label assignments (greater agreement between the atlases) have higher label agreements with manual labels. Therefore, the number of labels per voxel provides a rough confidence measure for each voxel's label. The error bars extend one standard deviation about the mean. Remaining data for voxels with five or more labels are not included for clarity.

Even with this dependence of absolute results on parcellation scheme, we may determine whether there is a relative improvement of results across all subjects as a function of the number of atlases used to obtain the voxelwise majority labels. From Table 1 and the accompanying graph in Figure 8, we may see that increasing the number of atlases asymptotically increases mean label agreement with manual labels. A one-way ANOVA was performed to test if the means are the same for the label agreements obtained by the different numbers of atlases. A multiple comparison test was then performed using Tukey's honestly significantly difference criterion to determine which pairs of means are significantly different. We see from Figure 9 that simply increasing the number of atlases from one to at least four results in a statistically significant increase in label agreement (*p *≪ 10^-6 ^for all comparisons), and further increasing the number of atlases to at least nine (or at least seven for the first set of subjects) results in a statistically significant increase in label agreement compared with using three atlases. However, the increase in label agreement from four to five or more atlases is not statistically significant for the mixed subject group.

One should not conclude based on these data that atlas databases need only contain four or five atlases to be representative. The standard deviations for our subject pool were high enough to warrant further investigation into sources of error. These sources include morphological dissimilarities between subject subpopulations, different parcellation schemes, and limitations of the Mindboggle algorithm. Interestingly, Kittler *et al*. [86] found that the classification performance of the voting rule applied to face and voice biometric data also peaked at four to five experts (atlases).

These results corroborate the conclusion of a study on atlas selection strategies applied to confocal microscopy images of bee brains, that labeling a brain image using every one of a group of atlases gives results superior to selecting an individual atlas [11]. However, when they tested the individual atlas condition, they chose only a single favorable atlas from a group of 20, whereas in the present study we ran tests using each and every single individual atlas from a group of 20.

The majority voting rule is probably not the optimal way to decide on a voxel's label [86-89], especially if the selected atlases deviate considerably from the subject brain to be labeled. A missing or unusual structure in a subject brain represented in only a minority of the atlases would most likely result in an inappropriate label. Rather than simply weighting the contribution of each of the atlases equally, each atlas vote for each subject voxel could be weighted by a function of the matching cost for the structure containing that voxel, since Mindboggle's matching cost function is intended to determine degree of correspondence between structures across brains.

We further separated the results by labeled region, to compare label agreement and type II errors between manual and Mindboggle labels for each label. As may be seen in Table 2, different numbers of atlases provide significantly higher label agreements for specific brain regions. Caviness *et al*. [12] found the percent label accord between two expert human labelers, the manual inter-rater reliability, to be 80.23% (*σ *= 8.08%) averaged across all 96 labels in four brains. Since we found, as did Caviness *et al*., a weak correlation between percent accord and region size, we should expect that a manual inter-rater reliability for our parcellation's fewer and larger regions to be somewhat higher than 80%. The problem with making a direct comparison between the same number and sizes of parcellation units is that Mindboggle relies solely on structural features to define anatomical boundaries whereas the Caviness approach also uses planes that extend far from the structural features used to construct the planes. We are presently evaluating Mindboggle on the entire set of 96 labels. The percent label accords obtained by Mindboggle in this study range in value across the different labeled regions, and average to 79.86% (*a *= 4.18%) for subject group 1 and 76.23% (*σ *= 5.17%) for subject group 2 (9 atlases for each subject), with the highest accords (> 90%) for the largest regions, the frontal and temporal poles and occipital lobes, and the lowest accords (< 70%) for the postcentral gyrii. The fact that the Mindboggle vs. manual accuracy is comparable to the reported inter-rater reliability is very encouraging.

For a single atlas to label a single subject, Mindboggle presently takes less than 17 minutes after linear registration and gray matter segmentation on a 2.2 GHz Pentium IV processor running Redhat Linux 9.0 on a PC with 1 GB memory: 1.3 minutes to construct a sulcus skeleton, 2.5 minutes to divide the skeleton with an interhemispheric plane, 3.4 minutes to construct and tally data on sulcus pieces, 2.5 minutes to find matching pieces in the atlas and to transform them from the atlas to the subject brain, and the remaining 7 minutes to warp and propagate labels through the gray matter mask. For each additional atlas, matching, warping, and labeling takes under 10 minutes if performed sequentially. For example, labeling a subject using five atlases would take 17 minutes if conducted in parallel, or an hour if conducted sequentially. The run time would reduce significantly not only by running Mindboggle for each atlas in parallel, but also by implementing faster preprocessing algorithms and optimized code rewritten in a lower-level language such as C as opposed to Matlab.

We conclude that by using multiple atlases, the overall label agreement between manual labels and the majority labels assigned by these atlases significantly improves when using a nonlinear procedure such as Mindboggle.

We are now in the process of applying this multiple atlas extension of Mindboggle to anatomically label functional activity data. Combining a confidence measure for anatomical boundaries derived from multiple atlases with statistical maps of functional activity data across subjects should help to establish our level of confidence in reported functional findings.

## Appendix

### Note 1

Since this study was conducted, Mindboggle no longer crops any part of the brain's gray matter; sulci are instead extracted by creating a mask by morphologically closing white matter (using Matlab's imclose.m function). This was visually determined to result in better sulcus extraction.

### Note 2

Mindboggle now splits the skeleton much more quickly and accurately with a surface constructed by selecting a medial slab of the skeleton, flattening the slab into a mean surface along the x-axis, and applying a median filter to the surface x values (Matlab's medfilt2.m).

### Note 3

Mindboggle no longer warps the atlas label volume or fills unlabeled regions by the majority label in its neighborhood. Instead, it simply fills the transformed boundaries with nearby labels according to a distance function (Matlab's bwdist.m).

## Competing interests

The author(s) declare that they have no competing interests.

## Authors' contributions

AK invented Mindboggle, conceived of and executed the study, and drafted the manuscript. BM, SG, and JT contributed manually labeled brain images. JH sponsored and supported the research. All authors read and approved the final manuscript.

## Pre-publication history

The pre-publication history for this paper can be accessed here:



## Figures and Tables

**Table 1:** Percent label agreement as a function of the number of atlases

A	All subjects	*σ*	II	Group 1	*σ*	II	Group 2	*σ*	II
1	74.39	(3.90)	0.20	79.05	(1.06)	0.17	73.55	(2.43)	0.21
2	73.75	(2.81)	0.21	78.38	(1.32)	0.18	72.68	(2.07)	0.21
3	76.54	(2.93)	0.19	80.19	(1.42)	0.17	75.30	(2.00)	0.20
4	77.73	(2.78)	0.18	80.95	(1.43)	0.16	76.31	(1.40)	0.19
5	77.88	(2.67)	0.18	81.98	(1.39)	0.15	77.24	(1.83)	0.19
6	78.30	(2.52)	0.18	81.58	(1.42)	0.16	77.45	(1.66)	0.18
7	78.56	(2.75)	0.18	82.09	(1.37)	0.15	77.76	(1.78)	0.18
8	78.67	(2.86)	0.18	82.21	(1.28)	0.15	77.87	(1.93)	0.18
9	79.18	(2.59)	0.17	82.50	(1.20)	0.15	78.15	(1.94)	0.18
10	78.91	(2.60)	0.17						
11	79.25	(2.75)	0.17						
12	79.47	(2.78)	0.17						
13	79.47	(2.86)	0.17						
14	79.41	(2.84)	0.17						
15	79.41	(2.83)	0.17						
16	79.48	(2.57)	0.17						
17	79.59	(2.74)	0.17						
18	79.61	(2.76)	0.17						
19	79.62	(2.74)	0.17						

Percent label agreements and type II errors (II) are given for each number of atlases (A) used to label subject group 1, group 2, and all subjects. The accompanying graph is in Figure 6. Standard deviations are in parentheses (for type II errors, *σ *equals 0.02 for all subjects and 0.01 for groups 1 and 2).

**Table 2:** Regions whose label agreement improves with multiple atlases

Labels	L	R
frontal pole		
sup. frontal		
mid. frontal		11
inf. frontal		
orbital	13	8
precentral		11
postcentral		
sup. parietal	8	9
inf. parietal		
temporal pole		4
sup. temporal	6	11
mid. temporal	17	12
inf. temporal	17	11
fusiform	7	11
lingual		
occipital lobe		
cingulate	9	
insula	9	3

The effect of multiple atlases on label agreement varies by manually labeled region. Here we compare label agreements obtained by Mindboggle for each region by different numbers of atlases randomly selected from either of the two subject groups (up to 19 atlases for each of the 20 subjects). Numerical entries denote the minimum number of atlases that result in significantly higher label agreements than for single atlas data, by region (*p *≪ 0.0001 for all regions except inferior temporal gyms: *p *< 0.01 and right temporal pole: *p *< 0.0002). For example, an entry of "9" means that significantly higher results were obtained using 9, 10, 11,... to 19 atlases versus using one atlas). The significance test is the same as that applied to the whole brain label agreement data.
